# Annotated checklist of the leech species diversity in the Maloe More Strait of Lake Baikal, Russia

**DOI:** 10.3897/zookeys.545.6053

**Published:** 2015-12-14

**Authors:** Irina A. Kaygorodova

**Affiliations:** 1 Irkutsk State University, 5 Sukhe Bator Street, 664003, Russia; 2 Limnological Institute, 3 Ulan-Batorskaya Street, 664033 Irkutsk, Russia

**Keywords:** Hirudinea, checklist, endemic, Maloe More Strait, Lake Baikal

## Abstract

In this paper, the very first checklist of the freshwater leeches of Maloe More Strait, a special part of Lake Baikal, is presented. It includes 14 free-living and parasitic species, of which four species belong to endemic Baikal genera – two species from *Baicalobdella* and one species each from *Baicaloclepsis* and *Codonobdella*. The checklist highlights six potentially new morphological species recorded for the first time in the area. The exact systematic position is stated for all leech species. Each species from the list is provided with information on taxonomic synonymy, data on its geographic distribution, and ecological characteristics. New species records are additionally provided with brief morphological characteristics and photos of their external morphology.

## Introduction

The Baikal leeches (Hirudinea) are one of the least studied groups of invertebrates due to underestimation of their role in aquatic ecosystems and intractability of their taxonomy. In fresh and brackish waters, some leeches serve as invertebrate predators while others are infamous for their ability to feed on the blood of either invertebrates or vertebrates. The first group includes macrophagous leeches. These leeches have a large size relative to other freshwater invertebrates and a high density in the littoral zone of reservoirs and lowland streams making them critical to fish nutrition. This role is obviously underestimated at present. This may be attributed to peculiarities of their habitation and consequently of their sample collection. Macrophagous leeches are rarely found in hydrobiological collections; therefore, their abundance has not been taken into account and their role in ecosystems has often been undervalued. The second group consists of parasitic forms, which constitute the main part of the leech diversity, and their role in ecosystems is absolutely different. Being epizoic parasites, they have relevance to transmission of bacterial and viral infections (e.g. Faisal and Schulz 2009, Faisal et al. 2011), as well as hematozoa including trematodes, cestodes and nematodes (Demshin 1975), and parasitic flagellates (Khan 1976, Khamnueva and Pronin 2004, Burreson 2007), which are considered to be pathogenic organisms for aquatic animals.

An exploration of the Baikal parasitic leech diversity was begun by pioneering 19^th^ century German zoologist Adolf Eduard Grube. *Clepsine echinulata* Grube, 1871 (now *Baicaloclepsis echinulata* (Grube, 1871)), *Piscicola torquata* Grube, 1871 (now *Baicalodbella torquata* (Grube, 1871)) and *Codonobdella truncata* Grube, 1873 may have been the first freshwater leeches recorded from Lake Baikal, but their host relationships had not been identified. Non-parasitic leeches of Lake Baikal were excluded from any scientific interest for a long time due to their belonging to the common Siberian faunal assemblage, and the scientific pursuit of unique endemic elements. Later, the famous Russian scientist Nikolai Livanow, studying Baikal samples, described the species *Protoclepsis tesselatoides* Livanow, 1902, which has some morphological differences from the Palaearctic *Protoclepsis tessulata* (Müller, 1774) (now *Theromyzom tessulatum*) parasitizing waterfowl. Next, the endemic “flat” leech *Torix baicalensis* Shchegolew, 1922 (now *Paratorix baicalensis*) was discovered by Shchegolew (1922) in collections from 1916, but the host remains unknown. No publications on Baikal leeches appeared for the subsequent 35 years until the description of a new endemic genus and species *Baicalobdella cottidarum* (Dogiel and Bogolepova 1957), found on cottoid fish. Subsequently, Lukin and Epstein (1960a) described a new genus (*Baicaloclepsis*) and a new species (*Baicaloclepsis grubei*), the first leech record from the Maloe More Strait. The same authors then created a new subfamily (Toricinae), which included the genus *Baicaloclepsis* Lukin et Epstein, 1960 and newly established *Paratorix* (Lukin and Epstein 1960b). Since, in addition to the above, the following papers on the Baikal leeches have been published: Dogiel et al. 1949; Epstein 1961, 1973, 1987; Lukin 1967, 1976; Finigenova and Snimschikova 1991; Kozhova and Izmest’eva 1998; Rusinek 2007. In order to revise the Lake Baikal leech fauna, the most recent target investigations have worked towards clarifying the taxonomic status of various species as well as adding to the species list with new records (Kaygorodova 2012, 2013; Kaygorodova and Natyaganova 2012; Kaygorodova and Utevsky 2012; Kaygorodova and Pronin 2013, Kaygorodova et al. 2013, Kaygorodova and Mandzyak 2014, Kaygorodova and Sorokoivikova 2014, Kaygorodova and Petryaeva 2014). Despite a recent surge of interest, the study of Baikal hirudinids is still in its infancy and, at the very least, there is a need for a provisional checklist as a starting point for further study. The present paper aims to provide such a list for the Maloe More Strait, a special part of Lake Baikal.

The data included here are based on previously published records and additional field investigations from 2002–2014. Collected material has been deposited in the laboratory of Molecular Systematics, Limnological Institute SB RAS, Irkutsk, Russia. The list provides morphologic and taxonomic notes where needed, as well as distribution ranges of genera and species. The accepted modern names of the type species of genera are provided. The systematic arrangement at family and more inclusive levels is based on the currently accepted classification system. Within this paper, family, subfamily, genus and species names are arranged alphabetically. The checklist includes 14 species and subspecies along with several new distribution records, including four endemic species, and six potential new species.

## Materials and methods

**Figure 1.**
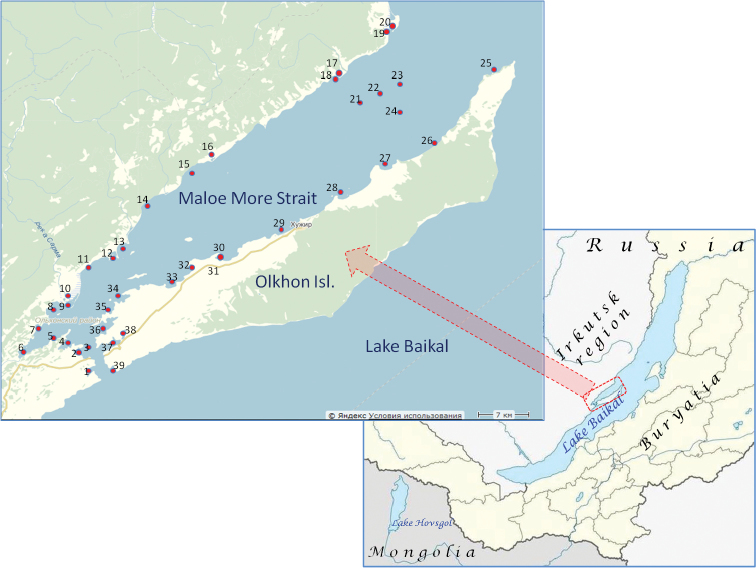
Map of the Maloe More Strait of Lake Baikal. Red dots indicate leech sampling localities: **1** Tutay Bay **2** Sakhurte Bay **3** Olkhon Gates Strait **4** Kurkut Bay **5** Ulirba Bay **6** Mukhor Bay **7** Shide Bay **8** Khuzhir-Nugho Bay **9** Khuzhir-Nugho Bay against of Sarma village) **10** Sarma River **11** Mukhor-Khale Bay **12** Khagden-Khale Bay **14** Kurma Bay **15** Otto-Khushun Bay **16** Lake Surkhaytor-Hur **17** Lake Zunduk **18** Zunduk Bay **19** Codoviy Bay **20** Lake Zama **21–24** Northern transit of the Maloe More **25** Cape Khoboy **26** Nyurgon Bay **27** Kharansa Bay **28** Odonim Bay **29** Khuzhir Bay **30** Elgay Bay **31** Lake Khankhoy **32** Shibetey Bay **33** Semisosennaya Bay **34** Khargoy Bay **35** Kharin Irgi Bay **36** Khul Bay **37** Zagli **38** Lake Nur **39** Ushun Bay.

Previously published information and an extensive collection of specimens, collected by the author in the period from 2002 to 2014 were used in this paper. Most of the specimens came from the task-oriented expedition on the Maloe More Strait which was undertaken in 2013. All sampling locations are shown in Fig. 1. Since the usual hydrobiological equipment (sweep net, dredge, scraper, bottom grab, etc.) is often less effective in procuring leeches than searching for many other aquatic invertebrates in order to catch parasitic and predatory leeches we inspected various aquatic plants and animals as well as different underwater objects (rotten wood, driftwood, snags, stones, etc.), to which hirudinids can be attached. Some leeches were picked out from zoobenthic samples. In most cases piscine and endemic flat leeches (glossiphoniids) were gathered directly in captured living hosts. Fish, molluscs and amphipods were collected by scuba divers at a depth of 2–42 m and by dredge or fishing gear at 30–200 m.

Newly collected specimens were photographed alive, placed in separate vials, fixed and kept in 80% ethanol solution. Current systematic keys (Lukin 1976, Epstein 1987, Nesemann and Neubert 1999) and several original taxonomical descriptions (Lukin and Epstein 1960a,b; Dogiel and Bogolepova 1957) were used for species identification. Morphological analysis was conducted using a stereomicroscope MSP-2 var. 2 (LOMO). All images were taken with a camera NIKON D700. All voucher specimens were deposited at the Laboratory of Molecular Systematics, Limnological Institute, Russia.

## Systematics

### Phylum Annelida Lamarck, 1809 Class Clitellata Michaelsen, 1919 Subclass Hirudinea Lamarck, 1818 (synonym Hirudinida)

#### Order Rhynchobdellea Blanchard, 1894

##### Family Glossiphoniidae Vaillant, 1890

###### Subfamily Glossiphoniinae Autrum, 1939

####### Genus Alboglossiphonia

Taxon classificationAnimaliaRhynchobdelleaGlossiphoniidae

Lukin, 1976

######## Geographic distribution.

Holarctic.

######## Type species.

*Alboglossiphonia heteroclita* (Linnaeus, 1761).

####### Alboglossiphonia heteroclita

Taxon classificationAnimaliaRhynchobdelleaGlossiphoniidae

(Linneaus, 1761)

Hirudo heteroclita : Linnaeus 1761; *Hirudo papillosa*: Braun 1805; *Hirudo trioculata*: Carena 1820; *Clepsine caranae*: Moquin-Tandon 1826; *Clepsine striata*: Apáthy 1888; *Clepsine polonica*: Lindenfeld and Pietruszynski 1890; *Glossiphonia heteroclita*: Blanchard 1894; *Glossiphonia heteroclita*: Harding and Moore 1926.

######## Geographic distribution.

Holarctic species.

######## Subspecies.

*papillosa* (Pawlowski, 1936)

######## Geographic distribution,

widespread in the Holarctic region.

Maloe More: Tutay Bay, Kurkut Bay, Zagli Bay, Mukhor Bay, Kurma Bay, Lake Zunduk, Lake Zama, Codoviy Bay, Lake Khuzhir.

######## Ecological characteristics.

*Alboglossiphonia heteroclita* lives in various types of flowing and stagnant waters (Nesemann and Neubert 1999). It has been recorded from brackish water, up to 3% in the Baltic Sea by Koli (1960). It occurs from the lowlands to mountainous regions. *Alboglossiphonia heteroclita* is a suctorial freshwater sit-and-wait predator; it preys on small invertebrates – mainly on gastropods, isopods and oligochaetes. It inhabits sor zones and warm bays of Lake Baikal. As a typical glossiphoniid, it shows touching parental care.

####### Alboglossiphonia hyalina

Taxon classificationAnimaliaRhynchobdelleaGlossiphoniidae

(Müller, 1774)

Hirudo hyalina : Müller 1774; *Clepsine hyalina*: Moquin-Tandon 1826; *Glossiphonia hyalina*: Blainville 1827; Glossiphonia heteroclita f. hyalina: Pawlowski 1936.

######## Geographic distribution.

Palaearctic region.

Maloe More: Kharin Irghi Bay, Kurma Bay, Shide Bay, Lake Khuzhir, Lake Khankhoy, Lake Surkhaytor-Nur, Lake Zunduk, Lake Zama, Codoviy Bay.

######## Ecological characteristics.

This species is a benthic ectoparasite of snails. It was found in the mantle cavity of representatives of Lymnaeidae, for instance *Lymnaea stagnalis* (Linnaeus, 1758) and *Stagnicola corvus* (Gmelin, 1791). In Baikal, *Alboglossiphonia hyalina* feeds on Planorbidae, Lymnaeidae, and Valvatidae.

####### Genus Glossiphonia

Taxon classificationAnimaliaRhynchobdelleaGlossiphoniidae

Johnson, 1817

######## Geographic distribution.

Palaearctic and Nearctic.

######## Type species.

*Glossiphonia complanata* (Linnaeus, 1758).

####### Glossiphonia sp. 1

Taxon classificationAnimaliaRhynchobdelleaGlossiphoniidae

######## New species records.

Kurma Bay, Lake Surkhaytor-Nur, Lake Zunduk, Lake Zama.

######## Morphological characteristics.

**Figure 2.**
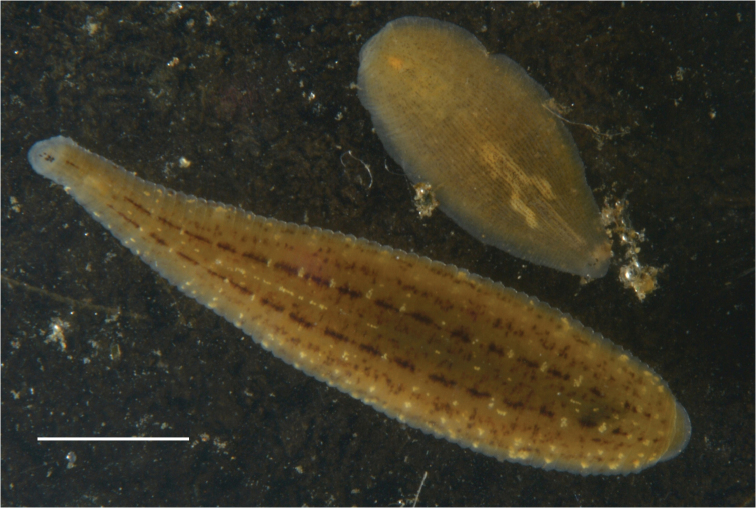
*Glossiphonia* sp. 1 (below) and *Alboglossiphonia heteroclita* (above) from Kurma Bay of the Maloe More Strait (Lake Baikal). Scale bar 10 mm.

Length is up to 25 mm. Three pairs of eyes. On the dorsal side there are longitudinal rows of dark pigmentation (Fig. 2). Central pair of stripes is always brighter in comparison with the more lateral ones.

######## Ecological characteristics.

Specimens were collected in the littoral zone. Life cycle is typical for majority of the genus. It prefers to sit on the rocks, or slowly crawl. This leech feeds almost exclusively on molluscs, and sometimes on worms or larvae of insects. With its elastic proboscis, it pierces the delicate covers of the victim and sucks its blood. The Maloe More *Glossiphonia* sp. 1, like other glossiphoniids, takes care of its young.

####### Glossiphonia sp. 2

Taxon classificationAnimaliaRhynchobdelleaGlossiphoniidae

######## New species records.

Kurma Bay, Lake Zama.

######## Morphological characteristics.

**Figure 3.**
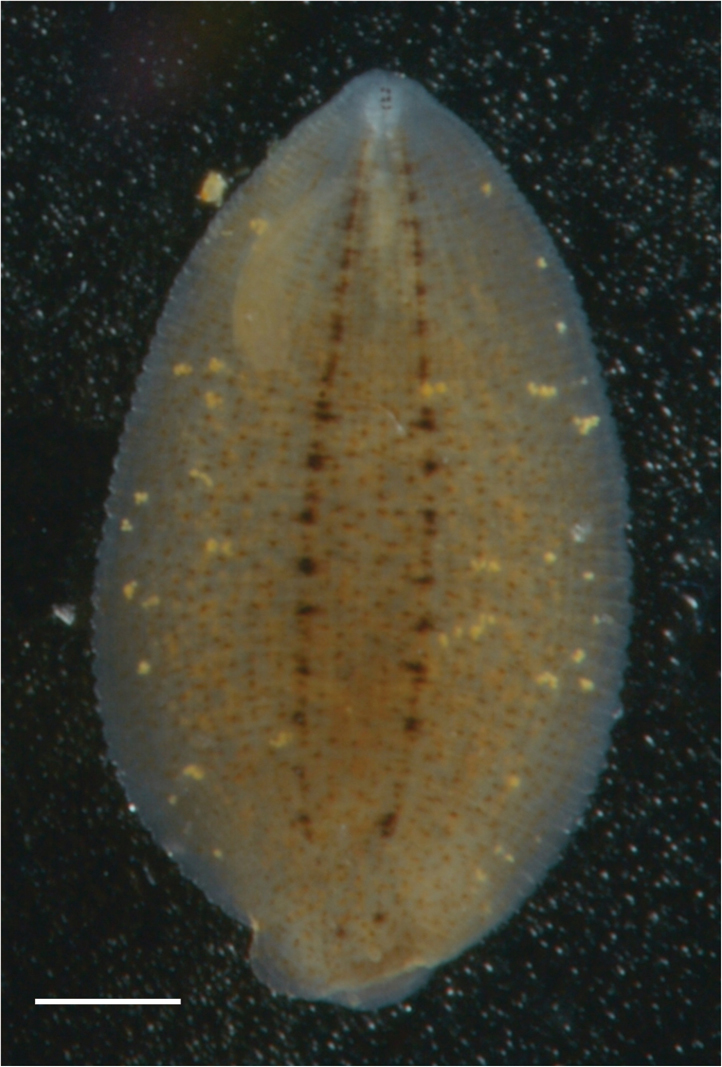
*Glossiphonia* sp. 2 from Kurma Bay of the Maloe More Strait (Lake Baikal). Scale bar 1 mm.

The size varies from 7 to 12 mm. This leech has a bright amber colour due to tiny pale brown pigment cells uniformly strewn along the body dorsally and one pair of dark longitudinal median rows (Fig. 3).

######## Ecological characteristics.

*Glossiphonia* sp. 2 occupies the same ecological niche as the previous *Glossiphonia* species, parasitizing small invertebrates preferentially molluscs.

####### Genus: Helobdella

Taxon classificationAnimaliaRhynchobdelleaGlossiphoniidae

Blanchard, 1876

######## Geographic distribution.

Cosmopolitan.

######## Type species.

*Helobdella stagnalis* (Linnaeus, 1758)

####### Helobdella stagnalis

Taxon classificationAnimaliaRhynchobdelleaGlossiphoniidae

(Linnaeus, 1758)

Hirudo stagnalis : Linnaeus 1758; *Hirudo pulligera*: Daudin 1800; *Glossiphonia perata*: Johnson 1816; *Erpobdella bioculata*: Lamark 1818; *Clepsine bioculata*: Savigny 1822; *Glossobdella pulligera*: Blainville 1827; *Clepsine stagnalis*: Fillipi 1837; *Glossiphonia bioculata*: Maquin-Tandon 1846; *Glossiphonia circulans*: Maquin-Tandon 1846; *Clepsine modesta*: Verrill 1972; *Glossiphonia modesta*: Vaillant 1890; *Glossiphonia stagnalis*: Blanchard 1894; Glossiphonia (Helobdella) stagnalis: Moore 1922; *Bakedebdella gibbosa*: Sciacchitiano 1939.

######## Geographic distribution.

Cosmopolitan.

Maloe More: Tutay Bay, Zagli Bay, Mukhor Bay, Shide Bay, Lake Khankhoy, Lake Surkhaytor-Nur, Lake Zunduk, Codoviy Bay.

######## Ecological characteristics.

This species is considered one of the most common freshwater leeches in the world. Within Baikal, *Helobdella stagnalis* inhabits shallow bays and salinas. This *Helobdella* species cannot swim; it crawls on aquatic plants and other objects, using its suckers as organs of attachment. Most suck the haemolymph of freshwater invertebrates such as oligochaetes, larvae of insects, and freshwater snails (Kozhova and Izmest’eva 1998). Freshwater jawless leeches are remarkable for their parental care. They produce a membranous bag or cocoon to hold the eggs, which are then carried on the ventral surface. The young attach to the parent’s belly after hatching and are thus ferried to their first meal.

####### Genus: Hemiclepsis

Taxon classificationAnimaliaRhynchobdelleaGlossiphoniidae

Vejdovský, 1884

######## Geographic distribution.

Palaearctic region.

######## Type species.

*Hemiclepsis marginata* (Müller, 1774)

####### Hemiclepsis marginata

Taxon classificationAnimaliaRhynchobdelleaGlossiphoniidae

(Müller, 1774)

Hirudo marginata : Müller 1774; *Hirudo variegates*: Braun 1805; *Hirudo cephalota* Carena 1820; *Hirudo oscillatoria*: Saint-Amas 1825; *Piscicola tesselata*: Maquin-Tandon 1826; *Piscicola linearis*: Kollar 1842; *Glossobdella cephalota*: Blainville 1827; *Haemoharis marginata*: Filippi 1837; *Glossiphonia marginata*: Maquin-Tandon 1846; *Hirido flava*: Dalyell 1953; *Glossiphonia flava*: Johnston 1865; *Glossiphonia marginata*: Blanchard 1892.

######## Geographic distribution.

Palaearctic region. A closely related taxon *Hemiclepsis marginata asiatica* Moore, 1924, is known from Cashmere to Sumatra. Its relationship to the nominate subspecies is still doubtful.

Maloe More: Kurma Bay, Mukhor Bay, Khuzhir-Nugho Bay, Lake Khankhoy, Lake Surkhaytor-Nur, Lake Zunduk.

######## Ecological characteristics.

This species inhabits Europe and Asia. In Central Europe, however, it is rare, whereas in Eastern Siberia it is widespread. As a sanguivorous ectoparasite it feeds on fishes and amphibians. *Hemiclepsis marginata* is able to move actively. When not on a host, *Hemiclepsis marginata* usually is found beneath large stones in shallow water or on submerged macrophytes. It can be found in all types of freshwater habitats and often thrives in stagnant water, weedy ponds, and, less often, in streams.

###### Subfamily Theromyzinae Sawyer, 1986.

####### Genus Theromyzon

Taxon classificationAnimaliaRhynchobdelleaGlossiphoniidae

Philippi, 1867

######## Geographic distribution.

Holarctic.

######## Type species.

*Theromyzon pallens* Philippi, 1867

####### Theromyzon tessulatum

Taxon classificationAnimaliaRhynchobdelleaGlossiphoniidae

(Müller, 1774)

Hirudo tessulata : Müller 1774; *Hirudo tesselata*: Bosc 1802; *Nephelis tesselata*: Savigny 1822; *Erpobdella tesselata*: Fleming 1822; *Ichthyobdella tesselata*: Blainville 1828; Erpobdella vulgaris var. tesselatum: Blainville 1828; *Clepsine tessulata*: Müller 1844; *Glossiphonia tessulata*: Maquin-Tandon 1846; *Glossiphonia aecheana*: Thompson 1846; *Hirudo vitrina*: Dalyell 1853; *Glossiphonia vitrina*: Johnston 1865; *Theromyzon tessulatum*: Philippi 1867; *Hemiclepsis tesselata*: Vejdovsky 1883; *Glossiphonia tesselata*: Blanchard 1892; *Protoclepsis tesselata*: Livanow 1902.

######## Geographic distribution.

Palaearctic and Nearctic regions. A closely related taxon *Protoclepsis tesselatoides* Livanow, 1902 was synonymised in *Theromyzom tessulatum* (Lukin 1976). This pooling into the nominate species is still doubtful.

Maloe More: Lake Zunduk, Lake Zama.

######## Ecological characteristics.

This is a widespread but rare species. It can be found in warm bays of Baikal and adjacent freshwater reservoirs. It prefers stagnant water. Feeds on the blood of vertebrates. Most likely hosts might be fishes, water birds or amphibians.

####### Theromyzon sp.

Taxon classificationAnimaliaRhynchobdelleaGlossiphoniidae

######## New species records.

Ulirba Bay.

######## Morphological characteristics.

**Figure 4.**
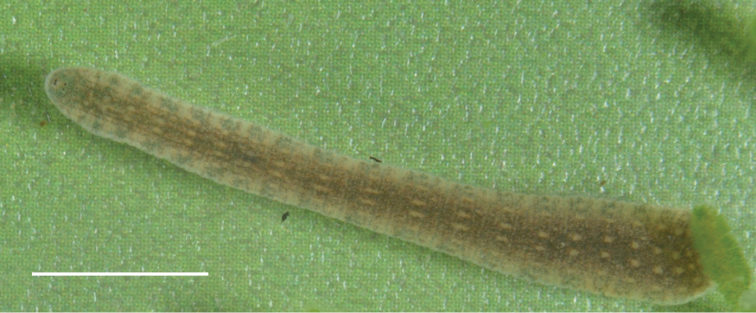
*Theromyzon* sp. individual inhabiting Ulirba Bay of the Maloe More Strait (Lake Baikal). Scale bar 5 mm.

Specimens are 12 mm in length and about 2 mm in width and can stretch up to 15-17 mm, becoming 1 mm in width. It has four pairs of eyes as do all representatives of the genus. A special colouration of the body sets them apart from all other known species (Fig. 4).

######## Ecological characteristics.

Representatives of this genus are known as bloodsuckers of birds (Sawyer 1986, Lukin 1976, Nesemann and Neubert 1999). The host for the Maloe More *Theromyzon* is unknown, since these specimens were found free-living.

###### Subfamily: Toricinae Lukin & Epstein, 1960

####### Genus Baicaloclepsis

Taxon classificationAnimaliaRhynchobdelleaGlossiphoniidae

Lukin & Epstein, 1960

######## Geographic distribution.

Endemic to Lake Baikal.

######## Type species.

*Baicaloclepsis echinulata* (Grube, 1871)

####### Baicaloclepsis grubei

Taxon classificationAnimaliaRhynchobdelleaGlossiphoniidae

Lukin & Epstein, 1960

Clepsine echinulata (part.): Grube 1871; *Haementeria echinulata*: Dogiel & Bogolepova 1957.

######## Geographic distribution.

Endemic to Lake Baikal.

Maloe More: Zagly Bay, Khargoy Bay, Semisosennaya Bay, Elgay Bay.

######## Ecological characteristics.

Endemic to Lake Baikal. Large leeches (length of 30-40 mm, width of 10-15 mm). *Baicaloclepsis grubei* were found only within the Maloe More Strait at relatively shallow depths of 14-40 m. This leech cannot swim and apparently can move only slowly. Since *Baicaloclepsis grubei* has a comparatively small posterior sucker, it is unlikely that it can provide a strong fastening to a host. In addition, this species probably spends a significant part of its life in a free-living state. All specimens were collected from benthic samples. The question of a potential host of this bloodsucking leech remains open.

##### Family Piscicolidae Johnston, 1865 (synonym Ichthyobdellidae Leuckart, 1863)

###### Subfamily Piscicolinae Caballero, 1956

####### Genus Baicalobdella

Taxon classificationAnimaliaRhynchobdelleaPiscicolidae

Dogel & Bogolepova, 1957

######## Geographic distribution.

Endemic to Lake Baikal.

######## Type species.

*Baicalobdella torquata* (Grube, 1871).

####### Baicalobdella cottidarum

Taxon classificationAnimaliaRhynchobdelleaPiscicolidae

Dogiel, 1957

Trachelobdella torquata (part.): Epstein 1959; *Trachelobdella torquata* (part.): Epstein 1959; *Trachelobdella torquata* (part.): Kozhov 1962; *Trachelobdella torquata* (part.): Lukin 1963; *Baicalobdella torquata* (part.): Lukin 1976.

######## Geographic distribution.

Endemic to Lake Baikal.

Maloe More: Kurma Bay; Olkhon Gates Strait; Kharansa Bay.

######## Ecological characteristics.

This species inhabits the littoral zone of Lake Baikal (0–200 m). This species is less abundant in the Maloe More area than its sister species, *Baicalodbella torquata*. In contradistinction to *Baicalodbella torquata*, it parasitizes only Baikal cottoid fishes. *Baicalobdella cottidarum* can be found directly on a host or in a free-living state on the surface of benthic substrates.

####### Baicalobdella torquata

Taxon classificationAnimaliaRhynchobdelleaPiscicolidae

(Grube, 1871)

Piscicola torquata Grube, 1871; *Trachelobdella torquata* (part.): Epstein 1959; *Trachelobdella torquata* (part.): Kozhov 1962; *Trachelobdella torquata* (part.): Lukin 1963.

######## Geographic distribution.

Endemic to Lake Baikal.

Maloe More: Khagden-Khale Bay; Otto-Khushun Bay; Nyurgon Bay; Odonim Bay; Ulirba Bay; Mukhor Bay; Sakhurte Bay; Khul Bay; Ushun Bay.

######## Ecological characteristics.

This is a typical component of the littoral zone of open water in Baikal. This species was found at depths of 0.5–10 m. These small leeches are 5-8 mm in length, with a width of 2-3 mm. Body colour varies from light green to pale rust, retaining a characteristic mosaic pattern on the dorsal side of the urosome. *Baicalobdella torquata* feed on Baikal endemic amphipods.

####### Genus Codonobdella

Taxon classificationAnimaliaRhynchobdelleaPiscicolidae

Grube, 1873

######## Geographic distribution.

Endemic to Lake Baikal.

######## Type species.

*Codonobdella truncata* (Grube, 1873).

####### Codonobdella sp.

Taxon classificationAnimaliaRhynchobdelleaPiscicolidae

######## New species records:

Northern transit of Maloe More Strait, Nyurgon Bay, Kharansa Bay, opposite the Cape Khoboy, Shibetey Bay.

######## Morphological characteristics.

**Figure 5.**
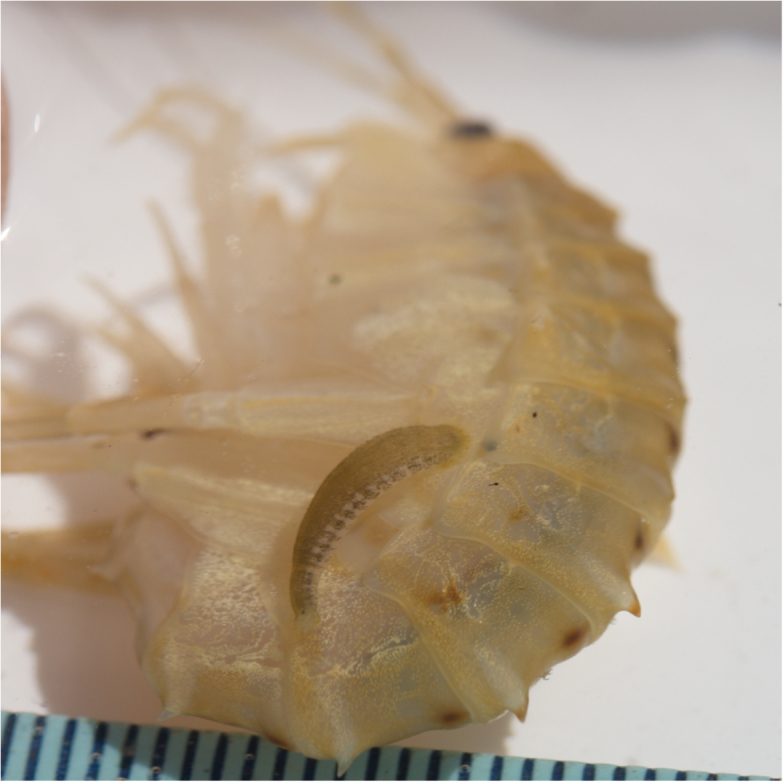
*Codonobdella* sp. on its host amphipod. The sample was found in the northern transit of the Maloe More Strait.

Body length of 8–10 mm. It differs from the type species, *Codonodbella truncata*, by the existence of a distinctive pigmentation on the dorsal side and the representative shape of the body (Fig. 5). There is a monotonous gray-green coloration of the dorsal side and a lighter colour on the ventral surface. A striped pattern is located laterally on each side of the body. Formerly, this leech was mistaken for *Piscicola geometra* (Kozhova and Izmest’eva 1998, Rusinek 2007) because of the similarity in colour patterns, the piscicola-like body shape and the lighter coloration of the ventral surface.

######## Ecological characteristics.

This leech is an inhabitant of open waters. Within the Maloe More, it was recorded in the northern part of the strait at a depth of 30–140 m.

#### Order Arhynchobdellea Blanchard, 1894  Suborder Erpobdelliformes Sawyer, 1986

##### Family Erpobdellidae Blanchard, 1894

###### Genus Erpobdella

Taxon classificationAnimaliaArhynchobdellidaErpobdellidae

de Blainville, 1818

####### Geographic distribution.

Palaearctic and Nearctic regions.

####### Type species.

*Erpobdella octoculata* (Linnaeus, 1758).

###### Erpobdella sp. 1

Taxon classificationAnimaliaArhynchobdellidaErpobdellidae

####### New species records.

Kurkut Bay, Kurma Bay, Zagli Bay, Shide Bay, Kharin Irghi Bay, Tutay Bay, Ulirba Bay.

####### Morphological characteristics.

**Figure 6.**
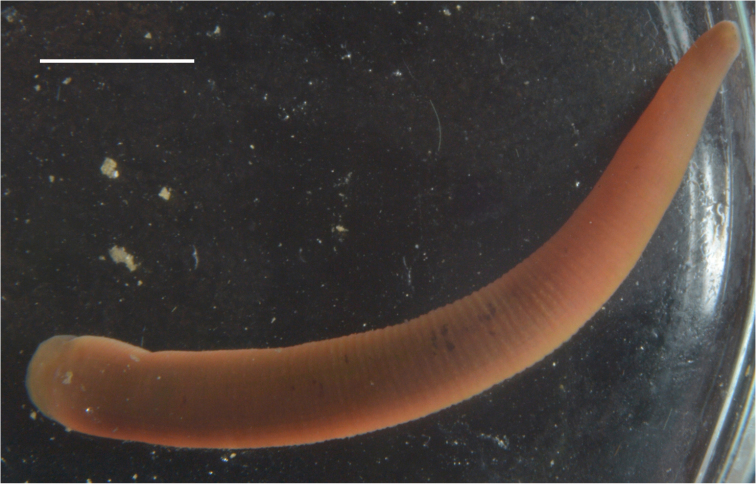
*Erpobdella* sp. 1 from Zagli Bay of the Maloe More Strait (Lake Baikal). Scale bar 5 mm.

The leeches are about 25–35 mm in length and 3–4 mm in width. Eight eyes. The leech has a pale pink body tinge. Dorsal pigmentation is almost absent. There are only a few dark spots irregularly scattered on the dorsal surface (Fig. 6).

####### Ecological characteristics.

This freshwater ribbon leech is common in the sor zone of Lake Baikal (Kaygorodova 2012). It was found mainly in the south-western part of the Maloe More. This is a non-parasitic leech. With a powerful pharynx *Erpobdella* completely or partially ingests different aquatic animals, including small annelids, crustaceans, insect larvae, molluscs, and even young fishes. In addition, it has been known to feed on dead animals and smaller specimens of its own species.

###### Erpobdella sp. 2

Taxon classificationAnimaliaArhynchobdellidaErpobdellidae

####### New species records.

Lake Zama, Lake Zunduk, Codoviy Bay.

####### Morphological characteristics.

**Figure 7.**
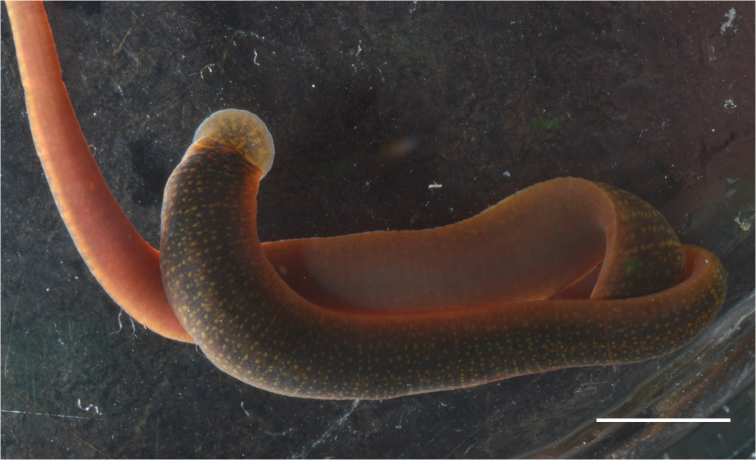
*Erpobdella* sp. 2 from Codoviy Bay (Maloe More Strait, Lake Baikal). Scale bar 5 mm.

These large sized leeches are up to 90 mm in length and 4.5–5.0 mm in width. The leeches have dark green or brown dorsal pigmentation flecked with yellow. Ventral pigmentation is almost absent (Fig. 7).

####### Ecological characteristics.

This non-parasitic macrophagous leech species has a restricted distribution even within the Maloe More. It was found only in lakes and bays of the most north-western coast of the strait.

## Conclusions

This is the first comprehensive checklist of the Baikal leech species inhabiting the Maloe More Strait. At present, 14 species are documented. This species diversity includes both widespread Holarctic and Palaearctic leeches and also endemic leech species from two different orders (Rhynchobdellida and Arhynchobdellida), three families (Glossiphoniidae, Piscicolidae, and Erpobdellidae) and nine genera. The most diverse is the group of glossiphoniid leeches, which consists of nine species belonging to six genera (*Alboglossiphonia* – 2 spp., *Glossiphonia* – 2 spp., *Helobdella* – 1 sp., *Hemiclepsis* – 1 sp., *Theromyzon* – 2 spp., *Baicaloclepisis* – 1 sp.). The Maloe More piscine leeches include representatives of two endemic genera (*Baicalobdella* – 2 spp. and *Codonobdella* – 1 sp.). Among the Arhynchobdellida, two species of the genus *Erpobdella* were found in the Maloe More. Six species in the checklist, including both representatives of *Erpobdella*, two of *Glossiphonia*, as well as one each from *Theromyzon* and *Codonobdella* were referred in this paper, with caution, to unidentified species since their morphology differed from all currently described species. With high probability, these six non-identified species are potentially new to science. All six of these, for the first time were recorded within the Maloe More. Some of these new morphotypes had previously been found in other parts of Lake Baikal. Thus, leeches similar to *Erpobdella* sp. 1 had already been reported from Chivyrkuy Bay (Kaygorodova 2012, 2013; Kaygorodova and Pronin 2013), whereas unidentified piscicolids *Codonobdella* sp. had been found throughout the lake (Kaygorodova 2012, 2013).

## Supplementary Material

XML Treatment for Alboglossiphonia

XML Treatment for Alboglossiphonia heteroclita

XML Treatment for Alboglossiphonia hyalina

XML Treatment for Glossiphonia

XML Treatment for Glossiphonia sp. 1

XML Treatment for Glossiphonia sp. 2

XML Treatment for Helobdella

XML Treatment for Helobdella stagnalis

XML Treatment for Hemiclepsis

XML Treatment for Hemiclepsis marginata

XML Treatment for Theromyzon

XML Treatment for Theromyzon tessulatum

XML Treatment for Theromyzon sp.

XML Treatment for Baicaloclepsis

XML Treatment for Baicaloclepsis grubei

XML Treatment for Baicalobdella

XML Treatment for Baicalobdella cottidarum

XML Treatment for Baicalobdella torquata

XML Treatment for Codonobdella

XML Treatment for Codonobdella sp.

XML Treatment for Erpobdella

XML Treatment for Erpobdella sp. 1

XML Treatment for Erpobdella sp. 2
